# Performance of entomopathogenic nematodes on the mealybug, *Dysmicoccus brevipes* (Hemiptera: Pseudococcidae) and the compatibility of control agents with nematodes

**DOI:** 10.21307/jofnem-2021-020

**Published:** 2021-02-25

**Authors:** Marcelo Zart, Mariana Ferracim de Macedo, Jael Simões Santos Rando, Gabriela Souza Doneze, Cassia Pereira Brito, Rodrigo de Souza Poletto, Viviane Sandra Alves

**Affiliations:** 1 Instituto Federal de Educação, Ciência e Tecnologia do Rio Grande do Sul, IFRS, Ibirubá, Rio Grande do Sul, Brazil; 2 Instituto Biológico de São Paulo, Campinas, São Paulo, Brazil; 3 Universidade Estadual do Norte do Paraná, Laboratório de Entomologia e Nematologia, Bandeirantes, Brazil; 4 Universidade Estadual do Norte do Paraná, Laboratório de Entomologia e Controle Microbiano (LECOM), Cornélio Procópio, Paraná, Brazil; 5 Universidade Estadual do Norte do Paraná, Laboratório Interdisciplinar de Pesquisa e Ensino de Botânica e Educação Ambiental (LIPEBEA), Cornélio Procópio, Paraná, Brazil

**Keywords:** Biological control, Pineapple mealybug, *Heterorhabditis amazonensis*, *Heterorhabditis indica*

## Abstract

The performance of nine isolates of *Heterorhabditis amazonensis* and one of *Heterorhabditis indica* on the mealybug *Dysmicoccus brevipes,* (Hemiptera: Pseudococcidae), were evaluated. The most virulent isolates were evaluated for nematode vertical and horizontal dispersal, and for efficiency at concentrations of 0 (control), 25, 50, 75, and 100 infective juveniles (IJs)/cm^2^ on adult females of the insect. A compatibility assessment was also carried out with commercial products, registered or in the process of registration, for use in the cassava culture. The isolates that caused the highest mortality rate of *D. brevipes* were NEPET11 (93.8% ± 4.1) and IBCB-n40 (84.0% ± 8.1), both isolates of *Heterorhabditis amazonensis*, while the isolate NEPET11 was more virulent than IBCB-n40 at all concentrations evaluated. In the dispersal test, the NEPET11 isolate caused mortality in the mealybug at a depth of up to 20 cm and a horizontal displacement of 7.25 cm. In the compatibility test, the NEPET11 isolate exhibited reduced viability due to the products Poquer, Tiguer 100 EC, Actara 250 WG, and Gaucho FS. The insecticide Curyom 550 EC was the only one that reduced infectivity (reduction of 92%) and is the only product classified as moderately toxic, while all the others were classified as compatible based on E%.

The mealy mealybug *Dysmicoccus brevipes* Cockerell (Hemiptera: Pseudococcidae), popularly known as pineapple mealybug, is considered one of the main pests of pineapple (Lacerda et al., 2009) and has also been reported in cassava (*Manihot esculenta* Crantz) (Lorenzi et al., 2016).

In cassava, *D. brevipes* is associated with roots, similarly to reported by Guide et al. (2016) for *Dysmicoccus* sp., where insects feed directly on tuberous roots, reducing the accumulation of reserves and causing a delay in plant development (Pietrowski et al., 2010; Takahashi and Gonçalo, 2005).

Even in other cultures, the management of these insects is considered unsatisfactory, due to their underground their habits, which impedes control by most natural enemies and agricultural pesticides (Alves and Moino, 2009; Guide et al., 2016; Souza and Ribeiro, 2003).

However, underground scale insects that attack roots can be easy targets for entomopathogenic nematodes (EPNs) (Rhabditida: Heterorhabditidae and Steinernematidae), which are organisms found in the soil and used for the control of insects that remain at least one phase of their life cycle in this environment, as is the case of mealybugs of the genus *Dysmicoccus* (Alves et al., 2009a; Grewal et al., 2001; Lewis et al., 2006; Stuart et al., 1997).

Studies to assess the pathogenicity of EPNs on mealybugs of the genus *Dysmicoccus* demonstrate that EPNs have the potential to control these insects. *Dysmicoccus texensis* (Tinsley) (Hemiptera: Pseudococcidae) presented up to 100% mortality in laboratory conditions (Alves et al., 2009a; Andaló et al., 2004a) and 65% in field conditions (Alves et al., 2009b). For *Dysmicoccus vaccini* Miller and Polavarapu (Hemiptera: Pseudococcidae), mortality of 83% was observed in the laboratory (Stuart et al., 1997) and in a study with pineapple mealybug*, D. brevipes*, some isolated of EPNs caused mortality higher than 94% (Ferreira et al., 2015). According to the aforementioned studies, the isolates of the *Heterorhabditis* were more virulent on scale insects, demonstrating their efficiency against this group of insects.

Thus, this study aimed to evaluate the virulence of nine isolates of the species *Heterorhabditis amazonensis* (Andaló, Nguyen, and Moino Jr.) and one of *Heterorhabditis indica* (Poinar, Karunakar, and David) on *D. brevipes*, in addition to evaluate the effect of different concentrations and the displacement capacity of infectious juveniles (IJs) on adult females of the insect. The compatibility of the most promising isolate with chemicals already registered or in the process of registration for cassava culture was also evaluated.

## Material and methods

### Acquiring insects and entomopathogenic nematode isolates

The insects (females of *D. brevipes*) used in the experiment were collected in a commercial cassava area in the state of Santa Catarina and multiplied in the laboratory in tuberous cassava roots. Subsequently, the insects were acclimatized under controlled conditions (25 ± 1°C, RH: 70 ± 10% without photoperiod) and were gradually transferred to pumpkins of the hybrid cabotiá cultivar (*Cucurbita maxima* × *Cucurbita moschata*), according to the method of Alves and Moino (2009). Once the breeding was established, the insects were then used in laboratory tests.

The nematodes used in the tests were purchased from the research institutions EMBRAPA TRIGO (Passo Fundo, RS), the Federal University of Lavras (Lavras, MG), and the Instituto Biológico (Campinas, SP) (Table 1). In the laboratory, the isolates were multiplied according to the methodology described by Molina and López (2001), using last instar larvae of *Galleria mellonella* (L.) (Lepidoptera: Pyralidae).

**Table 1.** Isolates, species, and collection sites of *Heterorhabditis amazonensis* and *Heterorhabditis indica* used in the selection test for adult females of *Dysmicoccus brevipes* in laboratory conditions.

Isolate	Species	Reference	Collection location
RSC05	*H. amazonensis*	Andaló et al. (2006)	Benjamin Constant – AM – Brazil
GL (Alho)	*H. amazonensis*	Andaló et al. (2009)	Lavras – MG – Brazil
JPM4	*H. amazonensis*	Molina et al. (2005), Andaló et al. (2014)	Lavras – MG – Brazil
RSC 03	*H. amazonensis*	Andaló et al. (2006)	Benjamin Constant – AM – Brazil
JPM3	*H. amazonensis*	Molina et al. (2005), Andaló et al. (2014)	Lavras – MG – Brazil
IBCB-n40	*H. amazonensis*	Dell’Acqua et al. (2013)	Taboporã – SP – Brazil
IBCB-n46	*H. amazonensis*	Dell’Acqua et al. (2013)	Santo Antônio de Posse – SP– Brazil
IBCB-n44	*H. amazonensis*	Dell’Acqua et al. (2013)	Santa Adélia – SP – Brazil
NEPET11	*H. amazonensis*	Ribeiro (2018)	Palmeira das Missões – RS– Brazil
IBCB-n05	*H. indica*	Dell’Acqua et al. (2013)	Itapetininga – SP – Brazil

### Isolate selection

EPN isolates (Table 1) were multiplied according to the methodology mentioned above and kept in aeration in 1-liter Erlenmeyer flasks, for a maximum of one week before their use in the assays. Each treatment (10 isolates + control) was repeated five times totaling 55 parcels, which consisted of a 100 mL plastic cup containing 100 g of sterile sand (autoclaved) and a piece of pumpkin, of 3 cm² paraffined on the opposite side of the bark to prevent fungal growth. The insects (10 females) were placed on the bark, and then covered with sand. The nematode isolates were inoculated with the aid of a micropipette in aqueous suspension at a concentration of 3.3 × 10^3^ infective juveniles (IJs)/cup (equivalent to 100 IJs/cm² of cup surface), diluted in 10 mL of distilled water (10% of relative to the sand mass). In the control treatment, the glasses received only distilled water.

The cups were closed with perforated plastic lids and kept in a climate chamber with temperature control (25 ± 1°C) and in the dark. The evaluation took place five days after inoculation, when dead insects were counted, and visual infection with nematodes was confirmed by dissecting the insects under a stereomicroscope.

### Concentration test

The *H. amazonensis* isolates NEPET11 and IBCBn-40 showed greater virulence in the isolate selection test and thus were used in subsequent tests. The concentration test employed the same methodology as the selection test, evaluating the virulence of EPNs on females of *D. brevipes* at concentrations: 0 (control treatment), 25, 50, 100, and 200 IJs/cm², and the assay was conducted in a 2 × 5 factorial design, with two isolates and five concentrations.

### Dispersal test

Two tests were performed evaluating vertical and horizontal dispersal of the EPNs, using *H. amazonensis* (isolate NEPET11).

To evaluate vertical dispersal, columns were assembled with 750-mL plastic cups, totaling 30 cm in height (3 cups). The substrate used was autoclaved fine sand, previously moistened with distilled water at 10% of the weight, which was placed in the three stacked cups and with their bottom parts removed to form an interconnected column. To assess the dispersal of the EPNs, four pieces of paraffined pumpkin were placed, which were arranged in four depths (0, 10, 20, and 30 cm, in ascending order from the top to the bottom of the column). Each piece of pumpkin received five *D. brevipes* females. After assembling the column, the NEPET11 isolate was applied with a micropipette, on the top of the column (depth 0), at a concentration of 100 IJs cm² per surface area of the cups.

In the test to evaluate the horizontal dispersal, a PVC tube structure was used, which was cut to be 5 cm length with 14.5 cm in diameter. The substrate (paraffined pumpkin) containing the insects (10 females) was prepared in the same manner as in the previous test and arranged in four different positions correspond in the circle N, S, E, and W (about North, South, East, and West) arranged around the perimeter, at an angle of 90 degrees. The nematode isolate was applied, at the concentration 100 IJs/cm² (1.65 × 10^4^ IJs/plot) in a suspension volume of 5 mL at the center.

In both dispersal tests, each treatment was repeated four times and the mortality of the mealybugs was evaluated five days after application. In both methodologies, the mortality was compared with a control treatment, which received only distilled water. The mortality of mealybugs was confirmed by dissection using a stereomicroscope.

### Test of compatibility with phytosanitary products

For the compatibility test, phytosanitary products were registered and/or under study for registration in the cassava culture (Table 2). The doses were calculated according to the manufacturer’s recommendation, with a solution volume equal to 144 liters per hectare and the isolate used was the NEPET11 (*H. amazonensis*).

**Table 2.** Trade name, biological activity, active ingredient, and dose of commercial product of phytosanitary products registered or in the process of registration for cassava culture, evaluated for compatibility with *Heterohabditis amazonensis* (isolated NEPET11) (AGROFIT, 2020).

Commercial name	Biological activity	Active ingredient	Situation	Dose/Ha^a^	Dose/L^b^
Curyom 550 EC	Insecticide	Lufenuron 50 g/L + profenofos 500 g/L	Registered	300 mL	2.08 mL
Gaucho FS	Insecticide	Imidacloprid 600 g/L	Not registered	360 mL	2.50 mL
Actara 250 WG	Insecticide	Thiamethoxam 250 g/kg	Not registered	150 g	1.04 g
Tiger 100 EC	Insecticide	Pyriproxyfen 100 g/L	Not registered	220 g	2.50 g
Nomolt 150	Insecticide	Teflubenzuron 150 g/L	Registered	300 mL	1.50 mL
Cipermetrina 250EC	Insecticide	Cypermethrin 250.0 g/L	Registered	50 mL	2.08 mL
Standak Top	Insecticide Fungicide	Pyraclostrobin 25 g/L + Thiophanate-methyl 225 g/L + Fipronil 250 g/L	Not registered	360 mL	0.69 mL
Poquer	Herbicide	Clethodim 240g/L	Registered	400 mL	2.77 mL
Fusilade 250 EW	Herbicide	fluazifop-butyl 250 g/L	Registered	700 mL	4.84 mL
Gamit 360 CS	Herbicide	Clomazone 360.0 g/L	Registered	2,000 mL	13.83 mL
Callisto	Herbicide	Mesotrione 480.0 g/L	Not registered	400 mL	2.77 mL

**Notes:** ^a^Grams (g) or milliliters (mL) of the commercial product per hectare; ^b^grams (g) or milliliters (mL) of the commercial product per liter (L) of distilled water.

Compatibility was assessed based on the adaptation of the IOBC/WPRS protocol, proposed by Vainio (1992). For this purpose, product mixtures were prepared at twice the dose indicated by the manufacturer (Table 2). Then, 1 mL of this solution was added to flat-bottomed glass tubes, and 1 mL of the nematode suspension in distilled water was also added, at a concentration of 2000 IJs/mL, resulting in 2 ml of solution at the concentration indicated by the manufacturer with 1,000 IJs/mL. A control treatment was carried out using only the suspension of EPNs in distilled water at a concentration of 1,000 IJs/mL. Each treatment was repeated five times and the experiment was conducted in a completely randomized design. The tubes were kept in a climatic chamber at 22 ± 1°C and 14 hr of photophase, for 48 hr, when the viability and infectivity were evaluated.

The viability assessment was carried out after 48 hr. After shaking the suspension, five 50 µL aliquots were removed from each tube and individually transferred to Elisa well plate. The number of live and dead IJs was counted until the total of 100 IJs, considering dead individuals those who did not move when touched with a probe.

To assess the infectivity, 3 mL of distilled water were added to each tube, stirred, and left to decant for 30 min at 10°C in the refrigerator. Then 3 mL of the supernatant was discarded, and the procedure repeated three times to eliminate product residues. After the third wash, five 0.2 mL aliquots were removed from the suspension of each tube and applied individually in Petri dishes (9 cm in diameter), containing two filter papers and 10 last instar larvae of *G. mellonella*. After five days the mortality of the larvae was evaluated.

### Statistical analysis

All experiments were repeated in time. The data from the selection test, different concentrations, displacement, as well as the viability and infectivity of the compatibility test were subjected to analysis of variance, and if the assumptions for homoscedasticity and normality were met, the means were compared by the Tukey test (*p* ≤ 0.05), through the statistical software SISVAR (Ferreira, 2019). The concentration test data were also submitted to a regression curve with the aid of the Excel program.

Phytosanitary products were also classified based on the value of E%, based on the formula prepared by Peters and Poullot (2004), but modified in this work, since production values were not considered. Thus, E% was calculated using the formula:

E%=100–(100–Mc%−Rinf%),

where Mc% refers to the mortality values of the viability test, obtained by the formula:

$$\mathrm{Mc}\%=\frac{\mathrm{Mo}\%\;-\mathrm{Mt}\%}{100-\mathrm{Mt}\%}\times 100,$$

where Mc% is the corrected mortality; Mo% the observed mortality; and Mt% the control mortality (control).

And Rinf% refers to the reduction of infectivity in treatments, which was determined by the formula:

$$\mathrm{Rinf}\%=\;\left(1-\;\frac{\mathrm{It}\%}{\mathrm{Ic}\%}\right)\times 100,$$

where Rinf% is the infectivity reduction; It% the infectivity of treatment; and Ic% the infectivity of control (control).

Based on the value of E%, the products were classified as: 1 – harmless (< 30%); 2 – slightly toxic (30-79%); moderately toxic (80-99%), and 4 – toxic (>99%).

## Results and discussion

### Isolate selection test

All isolates evaluated showed pathogenicity and caused confirmed infection in females of *D. brevipes*, with mortality values ranging from 46% (±6.0) to 93.8% (±4.1), with isolates IBCBn-40 and NEPET11, both of *H amazonensis* species were the most virulent, causing 84.0 and 93.8% of mortality, respectively (Table 3). Guide et al. (2016), evaluating the performance of EPNs on *Dysmicoccus* sp., a species of mealybug similar to *D. brevipes*, observed similar mortality rates for *H. amazonensis* isolates, with emphasis on NEPET11, IBCBn-10, and RSC05.

**Table 3.** Mortality (%) of *Dysmicoccus brevipes* female (mean ± SD) caused by isolates of *Heterorhabditis* in laboratory conditions (25 ± 1°, UR 70 ± 10% and without photophase).

Isolate	Species	% Mortality
Control	–	0.0 ± 0.0 a^a^
RSC05	*H. amazonensis*	46.0 ± 6.0 bc
IBCB-n46	*H. amazonensis*	48.5 ± 10.3 bc
IBCB-n44	*H. amazonensis*	48.9 ± 20.1 bc
IBCB-n05	*H. indica*	52.5 ± 6.5 bc
JPM4	*H. amazonensis*	54.0 ± 8.7 bc
JPM3	*H. amazonensis*	54.7 ± 6.3 bc
RSC 03	*H. amazonensis*	59.4 ± 7.4 cd
GL (Alho)	*H. amazonensis*	61.0 ± 14.8 cd
IBCB-n40	*H. amazonensis*	84.0 ± 8.1 de
NEPET11	*H. amazonensis*	93.8 ± 4.1 e

**Note:** ^a^Means followed by the same lowercase letter in the column did not differ from each other according to the Tukey test (*p* ≤ 0.05).

The isolate of the species *H. indica* (IBCBn-05) used in this study caused 52.5% mortality, which gave the lowest infection rate than the isolates of the species *H. amazonensis*, and was therefore discarded in subsequent tests. However, in a study developed by Ferreira et al. (2015), evaluating other isolates of EPNs on *D. brevipes* at different temperatures, the best results were obtained for isolates of the species *H. indica* (LPP22, LPP30, and LPP35) and *Heterorhabditis mexicana* Nguyen, Shapiro-Ilan, Stuart, McCoy, James & Adams (Hmex) when applied in the same conditions as the present work.

In the present study, the isolates that showed promise for the control of *D. brevipes* (NEPET11 and IBCB-n40) belongs *H. amazonensis.* Nevertheless, significant differences were observed for isolates RSC05, IBCBn-46 and 44, JPM3 and JPM4, of the same species.

Virulence variability for isolates from different species of nematodes on the same host was expected (Lewis et al., 2006), and has already been reported in the work of Ferreira et al. (2015) for *D. brevipes* and other similar species of mealybugs Alves et al., 2009a; Andaló et al., 2004a; Guide et al., 2016). The factors that may contribute to the difference in interspecific virulence include morphological differences, behavioral habits (foraging strategy), co-evolution with the host insect, as well as the various factors that involve the symbiotic nematode-bacteria complex during the infection process (Alonso et al., 2018; Shapiro-Ilan et al., 2017; Simoes and Rosa, 1996).

Intraspecific variation was also expected (Simoes and Rosa, 1996) and reported for other species of nematodes (Campos-Herrera and Gutierrez, 2009). Although less clear as to its origin, this variation may be related to the characteristics of the isolate, biotic, and abiotic factors of the soil from which they were found, and the host species with which they had contact during the evolutionary process (Shapiro-Ilan et al., 2006).

A factor that may have contributed to the differences in virulence observed in the present study is that the *H. amazonensis* isolates are from different regions of Brazil and co-evolved with different host species, which may have contributed to the development of host-pathogen specificity, resulting in different virulence against *D. brevipes*.

### Concentration test

**Figure 1:**
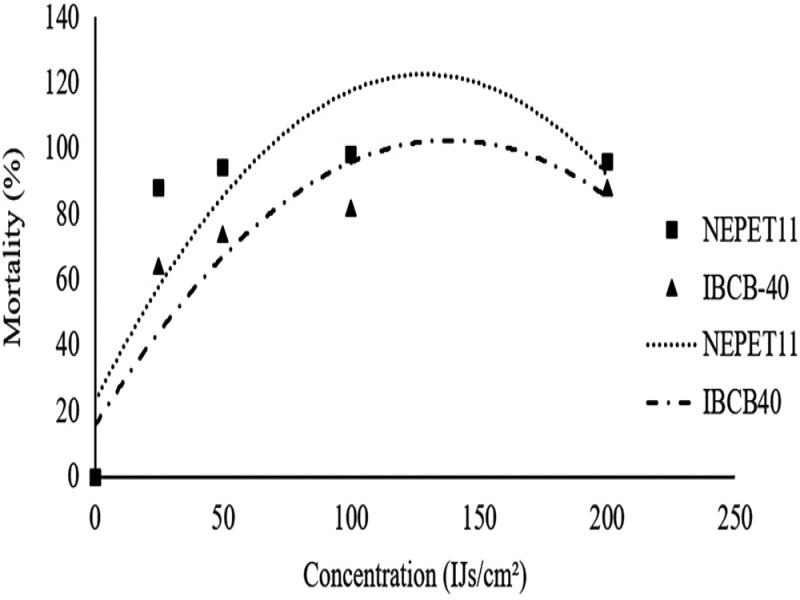
Mortality (%) from *Dysmicoccus brevipes* adult female (mean ± SD) caused by entomopathogenic nematodes applied in different concentrations in laboratory conditions (25 ± 1°, RH 70 ± 10% and without photophase). *Regression equations: NEPET11: *y* = −0.006 *x* ^2^ + 1,5478*x* + 22,985. *R* ^2^ = 0.732; IBCB40: *y* = 0.0045 *x* ^2^ + 12,568*x* + 15.6. *R* ^2^ = 0.8257.

Both isolates of *H. amazonensis* were pathogenic at all concentrations evaluated. However, NEPET11 was more virulent, causing 88% mortality in the lowest concentration evaluated (25 IJs/cm^2^), and did not demonstrate any significant difference from the other concentrations evaluated (Table 4). The IBCB-40 isolate achieved a significant increase in mortality as the concentrations increased, ranging from 64% (25 IJs/cm^2^) to 88% in the highest concentration evaluated (200 IJs/cm^2^) (Fig. 1).

**Table 4.** Compatibility of the entomopathogenic nematode *Heterohabditis amazonensis* (NEPET11) exposed for 48 hours to phytosanitary products registered or in the process of registration for cassava culture (IOBC/WPRS Protocol) (Vainio, 1992).

Data	Treatment	Viability (%)^a^	Infectivity (%)	MC%	Rinf%	E%	Classification^b^
28/05/2015	Control	92.2 ± 0.6 a^a^	92 ± 3.7 a	–	–	–	–
	Curyom 550 EC	90.8 ± 1.2 a	8 ± 3.7 b	1.52	91.30	92.82	Moderately toxic
	Poquer	79.8 ± 3.1 b	90 ± 6.3 a	13.45	2.17	15.62	Innocuous
20/06/2015	Control	99.4 ± 0.2 a	86 ± 5.1 a	–	–	–	–
	Fusilade 250 EW	99.2 ± 0.4 a	62 ± 8.6 a	0.20	27.91	28.11	Innocuous
	Cipermetrina 250 EC	97.4 ± 0.6 b	80 ± 8.9 a	2.01	6.98	8.99	Innocuous
	Gamit 360 CS	99.0 ± 0.3ab	74 ± 9.3 a	0.40	13.95	14.36	Innocuous
	Nomolt® 150	99.2 ± 0.2 a	66 ± 6.8 a	0.20	23.26	23.46	Innocuous
	Callisto	98.0 ± 0.5 ab	90 ± 5.5 a	1.4	0.00	1.41	Innocuous
24/07/2015	Control	92.8 ± 0.9 a	86 ± 2.4 a	–	–	–	–
	Tiger 100 EC	76.6 ± 2.5 b	76 ± 2.4 a	17.46	11.63	29.08	Innocuous
	Standak top®	87.2 ± 1.7 a	72 ± 4.9 a	6.03	16.28	22.31	Innocuous
	Actara 250 WG	70.8 ± 1.9 b	86 ± 5.1 a	23.71	0.00	23.71	Innocuous
	Gaucho FS	71.2 ± 2.5 b	86 ± 2.4 a	23.28	0.00	23.28	Innocuous

**Notes:** Mc% = corrected mortality; Rinf% = infectivity reduction; E% = product effect. ^a^Means followed by the same lowercase letter in the column, according to the date of application, did not differ from each other according to the Tukey test (*p* ≤ 0.05); ^b^product toxicity classification by IOBC: 1 – innocuous (< 30%), 2 – slightly toxic (30-79%), 3 – moderately toxic (80-99%), and 4 – toxic (> 99%).

The NEPET11 isolate was more virulent, providing the same control index at the lowest concentration (25 IJs/cm^2^) as the IBCBn-40 isolate at the highest concentration used (200 IJscm^2^) (Fig. 1). In a similar study, Guide et al. (2016) observed that the NEPET11 isolate caused 100% mortality in *Dysmicoccus* sp. at a concentration of 10 IJs/cm^2^, and 95% mortality for the RSC05 isolate applied at 50 IJs/cm^2^.

The high virulence of heterorhabditids on mealybugs was also reported by Ferreira et al. (2015), where the authors observed that the *H. indica* (LPP30 and LPP22), *H. mexicana* (Hmex), and *Heterorhabditis baujardi* (LPP35) isolates, at concentrations of only 3, 5, 6, and 10 IJs/cm^2^, respectively, were lethal to *D. brevipes*, thus proving their efficiency.

Lethal concentration is directly linked to the virulence of the isolate, and may be due to factors related to the nematode and the host, such as the search strategy of the isolate, the way it penetrates and causes infection, as well as its ability to circumvent the host’s immune system. Obtaining effective control at low concentrations is important, as it may lower costs for production and application of the isolate in biological control programs (Ferreira et al., 2015; Guide et al., 2016).

On the other hand, low concentrations obtained in the laboratory may not be as efficient in the field, where several other factors can interfere with the nematode’s action. Thus, experiments in field conditions must be carried out to confirm these values.

### Dispersal test

**Figure 2:**
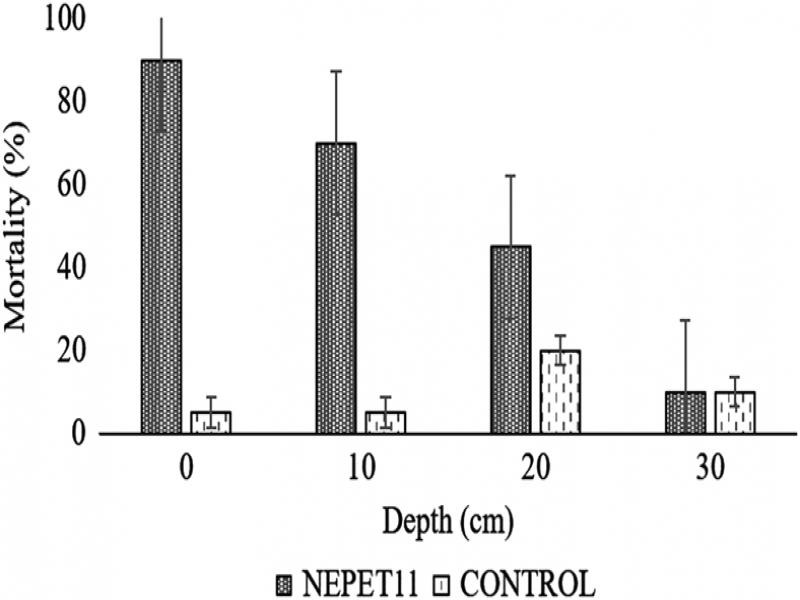
Mortality (%) from *Dysmicoccus brevipes* adult female (mean ± SD) caused by *Heterorhabditis amazonensis* (NEPET11) at different depths of sand column, under laboratory conditions.

In the vertical dispersal test, significant mortality of scale insects up to a depth of 20 cm was observed (Fig. 2). There was a significant reduction in mortality at depths of 10 and 20 cm (70 and 45%, respectively) in relation to the column surface, where mortality reached 90%. However, the dispersal of the EPNs up to 20 cm in the sand column, confirmed by the mortality of scale insects at this depth, means a favorable dispersal for possible use in the field, since the roots of the cassava culture are located mainly in the arable part of the soil, between 0 and 40 cm depth (Oliveira et al., 2001; Pequeno et al., 2007; Souza et al., 1994).

**Figure 3:**
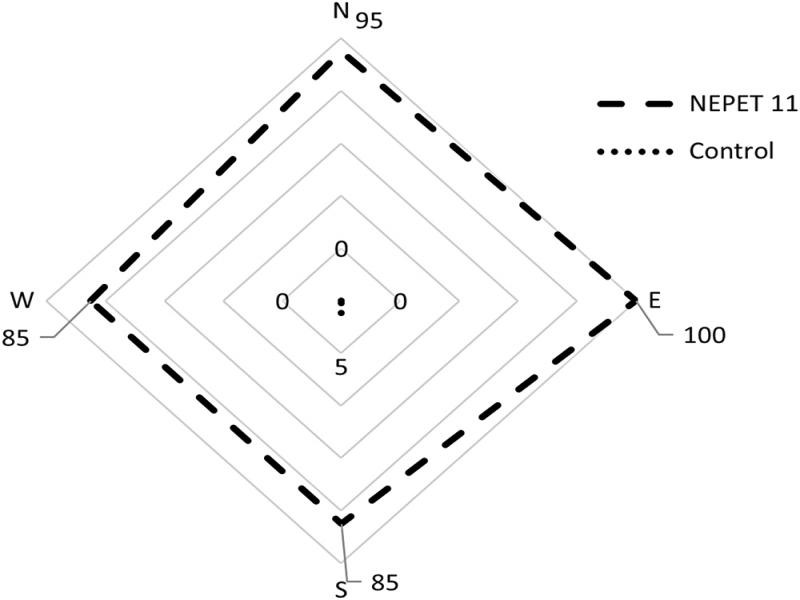
Mortality (%) of *Dysmicoccus brevipes* adult females (mean ± SD) caused by *Heterorhabditis amazonensis* (NEPET11) in a circular arena for observation of horizontal displacement.

For the horizontal dispersal test, mortality was observed in all evaluated positions, regardless of their disposition (Fig. 3), with values above 85%.

Guide et al. (2016) also observed vertical dispersal of the NEPET11 and RSC05 isolates in search on *Dysmicoccus* sp., and confirmed that the EPNs could efficiently controlling the cassava root mealybug at a depth of up to 5 cm. The results obtained in this work and in the work of Guide et al. (2016), who also evaluated the dispersal of isolates of the species *H. amazonensis*, indicate that it has a ‘cruizer’ habit, that is, it travels though the environment in search of hosts (Lewis et al., 1993). The type of search strategy and the ability of IJs to locate and infect hosts are important factors for effective biological control (Shapiro-Ilan et al., 2017).

In the field, the IJs move due to olfactory stimuli, released by the host (CO_2_ or other components secreted by the host) (Hallem et al., 2011; Dillman et al., 2012), volatile substances secreted by plants (Shapiro-Ilan et al., 2017), or even by identifying the host’s physical vibrations (Torr et al., 2004). Abiotic factors, such as temperature, humidity, pH, and soil type, can also interfere (Stuart et al., 2015). Although in this work, dispersal was observed only up to 20 cm, longer permanence in the environment or the presence of the host insect can serve as a stimulus for dispersal to greater depths.

### Test of compatibility with phytosanitary products

The compatibility test between the phytosanitary products and the NEPET11 isolate was performed at three different times, according to the dates recorded in Table 4. The products that reduced nematode viability were: Poquer^®^ (Clethodim) (20.2%), Tiger 100EC^®^ (Pyriproxyfen) (23.4%), Actara 250WG^®^ (Thiamethoxam) (29.2%), and Gaucho FS^®^ (Imidacloprid) (28.8%) (Table 4). The other tested products did not significantly affect the viability and presented values close to the controls.

The assessment of infectivity after exposure of the NEPET11 isolate to the products indicated that the insecticide Curyom reduced the percentage of infectivity on *G. mellonella* by 92%. The other products did not affect the infectivity of NEPET11.

Andaló et al. (2004b) evaluated the compatibility of phytosanitary products recommended for coffee cultivation with entomopathogenic nematodes (*Steinernema carpocapsae*, *Steinernema glaseri*, *Steinernema arenarium,* and *Heterorhabditis bacteriophora*). Only thiamethoxam significantly reduced the viability of *S. carpocapsae,* while imidacloprid had no effect on the isolates mentioned. However, the two products did not interfere in the nematode infectivity when inoculated on *G. mellonella* larvae, and thus were considered compatible based on the protocol by Vainio (1992).

Tavares et al. (2009) performed a compatibility test of *H. Indica* and *Steinernema* sp., using fipronil, thiamethoxam, and imidacloprid, confirming the compatibility of these biological agents with these insecticides. In a laboratory test, the mortality of EPNs ranged from 0.7 to 3% when exposed to imidacloprid. In a similar test, the nematode *H. amazonensis* JPM4 mixed with thiamethoxan, did not suffer interference in its viability and infectivity, demonstrating its compatibility for simultaneous use to control tomato larvae (Sabino et al., 2019). However, Negrisoli et al. (2008a, b) tested thiamethoxan on the viability and infectivity of the nematode *H. bacteriophora* and observed no compatibility, with a reduction in viability (88%) and infectivity (10%), but a much higher product dose (2 kg/ha) was used in that experiment than ours (0.15 kg/ha).

Some phytosanitary products from the chemical group of organophosphates, in which the Curyom^®^ product belongs, are recommended to control phytonematodes (AGROFIT, 2020). The active ingredient of this insecticide, profenofos, acts mainly by inhibiting acetylcholinesterase (enzyme that breaks down acetylcholine) (Santos et al., 2007), which affects the motor coordination of the organism, inhibiting the feeding and movement of contaminated individuals, which may have affected the ability of the NEPET11 nematode to cause infection in the host. Similar results have been reported for the isolate IBCB-n40, which after exposure to the insecticide carbofuran, exhibited no effect on viability, but its infectivity decreased (Bortoluzzi et al., 2013).

Thus, at the end of the compatibility test in this work, and according to the calculation of E%, only the product Curyom 550 EC was considered moderately toxic, with all the others being harmless to the NEPET11 nematode.

In addition to virulence data, other factors must be taken into account when aiming at the use of an entomopathogen in pest control programs, such as data on the nematode search habit, as well as the possibility of integration with the pesticides used in the culture, through compatibility studies between the EPNs and products used in agriculture. EPNs are generally compatible with some chemicals, which allows for joint use with chemical control methods (Andaló et al., 2004b; Negrisoli et al., 2008a, b).

In addition, the use of native isolates from regions near the pest area may be better adapted to the same conditions as the host, having developed specificity, thus, exerting greater virulence and pathogenicity even at lower concentrations, which is commercially interesting (Alves and Moino, 2009; Ferreira et al., 2015). These nematodes are also more advantageous from the environmental point of view compared to exotic species (Dolinski and Moino, 2006).

## Conclusion

The NEPET11 isolate of *H. amazonensis* was the most virulent on *D. brevipes* (caused 88% mortality in scale insects at a concentration of 25 IJs/cm^2^). This nematode was able to move vertically and horizontally in the soil. In addition, it was proven as compatible with most of the evaluated phytosanitary products.
